# Cardiac and Renal Fibrosis, the Silent Killer in the Cardiovascular Continuum: An Up-to-Date

**DOI:** 10.3390/jcdd11020062

**Published:** 2024-02-16

**Authors:** Traian Chiuariu, Delia Șalaru, Carina Ureche, Laura Vasiliu, Ancuta Lupu, Vasile Valeriu Lupu, Adela Mihaela Șerban, Alexandra Zăvoi, Laura Catalina Benchea, Alexandra Clement, Bogdan-Sorin Tudurachi, Radu Andy Sascău, Cristian Stătescu

**Affiliations:** 1 Department of Internal Medicine, Faculty of Medicine, Grigore T. Popa University of Medicine and Pharmacy of Iasi, 16 University Street, 700115 Iasi, Romania; traian.chiuariu@gmail.com (T.C.); laura.tapoi@yahoo.com (L.V.); alexandra.zavoi@gmail.com (A.Z.); benchea.lauracatalina@yahoo.com (L.C.B.); alexandram.clement@gmail.com (A.C.); bogdan-sorin.tudurachi@d.umfiasi.ro (B.-S.T.); radu.sascau@gmail.com (R.A.S.); cstatescu@gmail.com (C.S.); 2 Prof. Dr. George I.M. Georgescu Institute of Cardiovascular Diseases, Carol I Boulevard, No. 50, 700503 Iasi, Romania; 3 Department of Pediatrics, “Grigore T. Popa” University of Medicine and Pharmacy, 700115 Iasi, Romania; anca_ign@yahoo.com (A.L.); valeriulupu@yahoo.com (V.V.L.); 4 Cardiology Department, Heart Institute Niculae Stăncioiu, 19-21 Motilor Street, 400001 Cluj-Napoca, Romania; adelamserban@yahoo.com

**Keywords:** chronic kidney disease, heart failure, interstitial fibrosis, FGF23, Klotho

## Abstract

Cardiovascular disease (CVD) and chronic kidney disease (CKD) often coexist and have a major impact on patient prognosis. Organ fibrosis plays a significant role in the pathogenesis of cardio-renal syndrome (CRS), explaining the high incidence of heart failure and sudden cardiac death in these patients. Various mediators and mechanisms have been proposed as contributors to the alteration of fibroblasts and collagen turnover, varying from hemodynamic changes to the activation of the renin–angiotensin system, involvement of FGF 23, and Klotho protein or collagen deposition. A better understanding of all the mechanisms involved has prompted the search for alternative therapeutic targets, such as novel inhibitors of the renin–angiotensin–aldosterone system (RAAS), serelaxin, and neutralizing interleukin-11 (IL-11) antibodies. This review focuses on the molecular mechanisms of cardiac and renal fibrosis in the CKD and heart failure (HF) population and highlights the therapeutic alternatives designed to target the responsible pathways.

## 1. Introduction

Cardiovascular disease (CVD) is highly prevalent in patients with chronic kidney disease (CKD), determining a worse prognosis compared with the non-CKD population. On the other hand, CKD has been observed in 26–63% of HF patients, suggesting a bidirectional relationship in which both entities impact patient evolution and prognosis [1,2]. In the past two decades, cardio-renal syndrome (CRS), which comprises a spectrum of disorders in which an acute or chronic dysfunction of the heart or kidney results in the dysfunction of the other, has been recognized as a clinical entity. Encompassing five different types of CRS, this classification underlines the intertwined pathophysiological pathways and helps guide therapeutic decisions [3,4,5].

As CKD advances, several mechanisms contribute to increased fibrogenesis in organs and tissues, including the heart. It has been proposed that in CKD, various pro-fibrotic factors can induce biomechanical stress on cardiac cells and thus trigger myocardial interstitial fibrosis [6]. These factors include hemodynamic factors (pressure and volume overload) and non-hemodynamic factors (increased synthesis of fibroblast growth factor-23 (FGF-23), nitric oxide inhibitors, advanced glycation end-products, trimethylamine N-oxide, cystatin C, and endogenous cardiotonic steroids alongside the activation of the renin–angiotensin–aldosterone system) [7]. Angiotensin II (Ang II) and aldosterone have multiple effects that are important in the pathogenesis of the CRS, including activation of pathways associated with inflammation, fibrosis, extracellular matrix (ECM) accumulation, reactive oxygen species, and endothelial dysfunction [7,8].

This review provides an update on the molecular mechanisms of cardiac and renal fibrosis in the CKD and heart failure (HF) population, and highlights the therapeutic alternatives designed to target the responsible pathways.

## 2. Mechanisms and Molecules Involved in Activation and Promotion of Fibrosis

### 2.1. Renin–Angiotensin–Aldosterone System

In the setting of HF, RAAS and the sympathetic nervous system (SNS) are activated as compensatory mechanisms for the maintenance of extracellular volume homeostasis. However, these responses can lead to CKD progression in the long term. A study by Rafiq et al. on rats with uninephrectomy and surgically induced aortic regurgitation showed changes in the structure and function of the heart caused by chronic volume overload, with a corresponding increase in both SNS and RAAS activity. It was proposed that activation of the SNS and local Ang II stimulate reactive oxygen species generation in the kidney in a NADPH oxidase-dependent manner, which subsequently leads to podocyte injury and albuminuria [9].

Apart from its essential role in volume homeostasis, RAAS is a major contributor to cardiac and renal fibrosis development and progression via the transforming growth factor beta (TGFβ) signaling pathway. TGF-β1 promotes fibrosis via activation of both Smad-based and non-Smad-based signaling pathways, which result in the activation of myofibroblasts and excessive production of ECM. Additionally, TGFβ also signals a different, non-canonical pathway by activating multiple kinases, including the extracellular signal-regulated kinase (ERK), p38 mitogen-activated protein kinase (MAPK), and c-Jun N-terminal kinase (JNK) [10].

Aldosterone represents a significant contributor to cardiac and renal inflammation and fibrosis. Elevated aldosterone levels are responsible for interstitial and perivascular fibrosis within the myocardium, alongside glomerular impairment and tubulointerstitial fibrosis within the renal architecture. Additionally, aldosterone is associated with the recruitment of inflammatory cells and increased expression of inflammatory markers such as cyclooxygenase-2, monocyte chemoattractant protein 1, and intercellular adhesion molecule 1 across both cardiac and renal tissues. This cascade of inflammatory responses ultimately culminates in fibrotic remodeling. Besides the TGF-β1 activation, aldosterone also stimulates the expression of other molecular pathways with fibrotic potential: plasminogen activator inhibitor 1 (PAI-1), endothelin 1 (ET-1), placental growth factor (PGF), connective tissue growth factor (CTGF), osteopontin, and galectin-3 [11]. TGF-β1 and aldosterone were proven to increase PAI-1 expression and decrease extracellular degradation in rat mesangial cells and renal fibroblasts. PAI-1 promotes fibrosis and remodeling by preventing the activation of plasmin-mediated matrix metalloproteinase (MMP) and by reducing extracellular matrix degradation [12].

Furthermore, aldosterone increases ET-1 expression in the heart and vasculature, and subsequently, ET-1 increases collagen synthesis by cardiac fibroblasts in a TGF-β1-dependent manner. In vascular smooth muscle cells, aldosterone increases osteopontin expression by a mineralocorticoid-receptor [13] dependent mechanism involving ERK and MAPK. In the end, aldosterone increases the expression of galectin-3 in the heart, which stimulates collagen synthesis in cultured vascular smooth muscle cells, together with cardiac fibroblast proliferation, fibrosis, and left ventricular dysfunction [11].

In humans, the inhibition of RAAS represents the cornerstone of treatment all across the spectrum of cardio-renal disease. Data support the efficacy of RAAS inhibition in mitigating fibrosis, with clinical trials demonstrating that medications targeting this system significantly reduce fibrosis in various organs. In patients with heart failure, RAAS inhibition has been shown to decrease myocardial fibrosis, improving cardiac function and reducing adverse outcomes. Similarly, in patients with CKD, RAAS inhibition slows the progression of renal fibrosis, preserving kidney function and delaying the need for dialysis or transplantation [14].

### 2.2. Fibroblast Growth Factor 23 (FGF23) and Klotho Protein

FGF23 was first identified in the ventrolateral thalamic nucleus of the brain in the year 2000 as a new member of the fibroblast growth factor family. Later, it was observed that its highest expression resides in the osteocyte but that under pathological conditions, it can be secreted by the heart, liver, kidney, macrophages, or bone marrow [15,16,17].

FGF23 targets the kidney and parathyroid glands to regulate phosphate and vitamin D homeostasis [18]. In patients with CKD, hyperphosphatemia stimulates the FGF23 synthesis, inhibiting phosphate reabsorption by reducing the expression of the sodium/phosphate co-transporters NaPi-2a and NaPi-2c expression in the proximal tubules. Also, FGF23 inhibits the secretion of parathyroid hormone (PTH) by the parathyroid gland, downregulates 1a-hydroxylase (CYP27B1), and upregulates 24-hydroxylase (CYP24A1), which leads to the reduced synthesis and increased degradation of active vitamin D [17,19].

In patients with CKD, elevated FGF23 levels have been reported to induce impaired flow-mediated dilation, arterial stiffness, atherosclerosis, and left ventricular hypertrophy. Besides these pathological actions, evidence has recently emerged that FGF-23 is responsible for promoting cardiac fibrosis [19]. Moreover, in the last decade, several studies have pointed towards FGF23 as a non-traditional CV risk factor in patients with CKD, with an impact on CV and all-cause mortality [20,21].

In mice, Hao et al. investigated the role of FGF23 as a promoter of cardiac fibrosis, confirming that adult mouse cardiac fibroblasts secrete this protein and that the cells carrying the FGF23 gene after a myocardial infarction exhibit more fibrosis than control cells. These mechanisms seem to be related to the activation of β-catenin, which mediates the upregulation of TGF-β, collagen I, and collagen III, which leads to myocardial fibrosis [18]. Tomohiro et al. continued the research in this field using a deoxycorticosterone acetate (DOCA)-salt mouse model with mild chronic kidney disease, hypertension, and HF with preserved ejection fraction, which was treated with continuous intravenous FGF23. In the semiquantitative analysis, the fibrotic area was significantly larger in the group treated with FGF23 than in the control group treated with DOCA alone. Interestingly, the fibrosis area was significantly smaller in the group treated with DOCA, FGF23, and calcitriol, suggesting that calcitriol limited cardiac fibrosis by blocking the TGF-β—Smad 2/3 pathway and this effect was independent of the systemic and local levels of FGF23 [19].

In humans, a group by Lee et al. observed that FGF23 enhances human atrial fibroblast activity by activating FGF receptor 1 (FGFR1) and the subsequent phospholipase C/inositol 1,4,5-trisphosphate signaling pathway, which leads to the upregulation of calcium release-activated calcium channel protein 1 (Orai1) (mediated calcium (Ca^2+^) entry) and IP3 receptor (IP3R) (mediated Ca^2+^ release from endoplasmic reticulum), thus stimulating cardiac fibroblast proliferation and migration [22]. Moreover, another recent study demonstrated that expression of FGF23 is induced in cardiac myocytes by RAAS, and consequently, cross-talk between fibroblasts and cardiomyocytes occurs with pro-fibrotic effects. Thus, FGF23 could also be involved in cardiac fibrogenesis in a paracrine or autocrine manner besides the endocrine manner [23].

**Figure 1:**
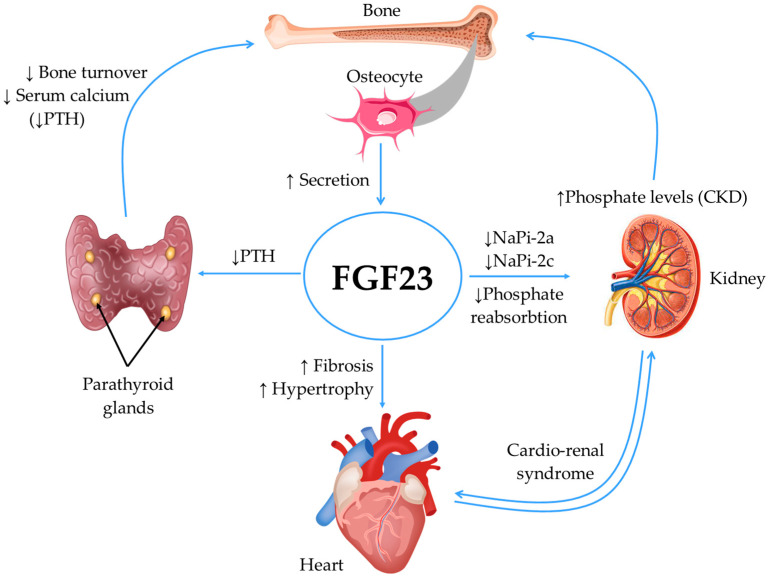
Mechanisms of action of FGF23. In CKD, hyperphosphatemia stimulates the FGF23 synthesis, which inhibits phosphate reabsorption and the secretion of PTH. This leads to reduced synthesis and increased degradation of active vitamin D. In the heart, high levels of FGF23 promote hypertrophy and fibrosis. CKD, chronic kidney disease; FGF23, fibroblast growth factor 23; NaPi-2a, sodium–phosphate cotransporter 2a; NaPi-2c, sodium–phosphate cotransporter 2c; PTH, parathormone.

The mechanisms of action of FGF23 are summarized in Figure 1.

Klotho is the co-receptor of FGFR1, implicated in the activation of the FGF23 signaling pathway [24]. Elevated circulating levels of FGF23 and Klotho deficiency are early metabolic consequences of CKD that are strongly associated with a greater risk of CVD events and mortality [19].

Klotho inhibits renal fibrosis, slows CKD progression, enhances mineral metabolism, ameliorates cardiomyopathy, and prevents vascular calcification by inhibiting TGF-β1 and Ang II [24]. Additionally, Klotho plays an important role in the progression of CVD by ameliorating myocardial hypertrophy induced by indoxyl sulfate in vivo. Also, a soluble form of Klotho produced from alternative splicing was shown to suppress myofibroblast proliferation and collagen synthesis in cultured mouse cardiac fibroblasts [23].

Important data come from Leifheit-Nestler et al., who examined myocardial autopsy samples of the left ventricle from dialysis patients and patients who had functioning renal allografts at the time of death. Using Klotho antibodies, they observed that the Klotho-positive area was significantly reduced in dialysis patients compared with matched controls. In addition, interstitial cardiac fibrosis was increased in dialysis patients but not those with a functioning renal allograft. The authors concluded that cardiac fibrosis is directly proportional to dialysis vintage and inversely proportional to the percentage of the Klotho-positive area in the myocardium of dialysis patients [23].

Klotho expression is often reduced in cardiovascular disease, correlating with disease severity and progression. By modulating oxidative stress, inhibiting inflammation, regulating vascular calcification, and improving endothelial function, Klotho exerts important cardioprotective effects in hypertension, coronary heart disease, and heart failure [25,26].

### 2.3. Kidney Injury Molecule-1 (KIM-1)

KIM-1 was first identified in 1996 as hepatitis A virus cellular receptor 1 (HAVcr-1), a membrane protein that facilitated the penetration of the hepatitis A virus into the cultivated kidney cells [27], with a high expression in the epithelial cells of proximal renal tubules in rats after induced ischemia [28]. This protein was also identified as TIM-1, a member of the TIM family (T-cell immunoglobulin and mucin domain family), expressed by the immune cells in numerous physiological and pathological processes [29,30]. This differentiates KIM-1 from the other members of the family, as it is present in both epithelial and immunocompetent cells. KIM-1 is expressed in all human tissues, but its highest levels are found in the kidney and testicles [31].

Renal ischemia [32] and hypoxia, as they are encountered in HF, represent a potent stimulus for KIM-1 expression in the cells of the proximal tubules, inducing chronic interstitial inflammation. Both the membrane-bound and free KIM-1 forms act in an autocrine and paracrine manner to mediate the interaction between the damaged proximal tubule cells and macrophages. Particularly, the interaction between KIM-1 and LMIR5/CD300b receptor on the resident myeloid cells promotes cytokine release chemoattractants for neutrophils. Afterward, local inflammation, hypoxia, and cell damage are enhanced, creating a positive feedback loop that leads to CKD progression accompanied by interstitial fibrosis [31]. Damman et al. showed that, in HF patients on diuretic treatment, KIM-1 is superior to other markers for unveiling subclinical tubular injury. Also, in patients with chronic HF or myocardial infarction, urinary KIM-1 is associated with an increased risk of death or hospitalization, independent of GFR [33,34].

The role of plasma levels of KIM-1 is still a matter of debate. In type I diabetic patients, elevated plasma levels of KIM-1 correlate with the reduction of glomerular filtration rate, albuminuria, and interstitial damage, suggesting that this glycoprotein could act as an early marker for CKD progression [31,32]. On the other hand, Egli et al. observed that plasma KIM-1 levels are not correlated with the markers of renal function impairment (creatinine and cystatin C), but they are strongly associated with CV risk factors: high arterial blood pressure, high low-density lipoproteins and C-reactive protein [35]. Recent data suggest that monitoring KIM-1 is also relevant for early diagnosis of kidney damage and clinical course in patients with CRS, HF, cardiopulmonary bypass, or cardiothoracic surgical interventions in the pediatric emergency setting [32].

Considering all of the above, KIM-1 is considered an important marker for kidney damage and a reliable predictor for acute kidney injury (AKI). Some authors nicknamed KIM-1 as “the renal troponin I” [32].

### 2.4. Neutrophil Gelatinase-Associated Lipocalin (NGAL)

NGAL was first identified in the granules secreted by neutrophils but has since been identified across a diverse range of cell types, including cardiomyocytes [36] and tubular epithelial cells [37]. NGAL is found in multiple isoforms (25 kDa, 45 kDa, and 145 kDa), but the 25 kDa isoform is almost exclusively produced in the kidneys [38]. It is a member of the lipocalin family, which mainly comprises proteins that transport lipophilic substances, with additional actions in cell division, differentiation, and adhesion [39].

NGAL is also recognized as being involved in the management of iron dynamics [40]. The iron-bound NGAL molecule engages with cell surface receptors, facilitating its entry into the cell, where iron is discharged afterward [41]. Conversely, free-of-iron NGAL can also bind to these cell membrane receptors, leading to iron mobilization from the cytoplasm to the interstitium [42].

NGAL is a useful marker for AKI, as it is released early in case of tubular damage [43,44]. When an ischemia-reperfusion episode occurs, a large amount of iron is released, leading to oxidative stress and tissue damage [45]. NGAL intervenes in iron homeostasis by binding to siderophores, allowing the regulation of intra- and extracellular levels of iron [46]. Taking into account that iron is important for bacteria development, NGAL also plays a bacteriostatic role by reducing iron availability [40,41].

Furthermore, Lee et al. emphasized the role of NGAL in inducing renal regeneration [47]. By injecting IL-10 overexpressing macrophages, NGAL secretion is promoted, and it helps with tissue repair and cell regeneration. This beneficial effect is lost when anti-NGAL antibodies are used [48], which emphasizes the protective role of NGAL in AKI.

Regarding CRS type 2, Angelini et al. described the complex interrelationship between the heart and the kidney as being a vicious circle. In these circumstances, the authors demonstrated that NGAL released by tubular cells binds MMP9 in both the heart and the kidney. In this respect, NGAL prevents MMP9 degradation, which increases its enzymatic activity and leads to enhancement of ECM degradation, worsening of heart remodeling, and further impact on kidney function [49]. Another study by Vescovo et al. demonstrated that cell therapy with human amniotic stem cells and vascular and stromal cells positively affects the remodeling of the heart and kidney. These results are explained by the fact that cell therapy decreased kidney NGAL production with a consequent reduction of the NGAL-MMP9 complex formation, which breaks the vicious cross-talk between the two organs [50].

In the spectrum of CVD, NGAL has been associated with the pathological mechanisms in HF, atherosclerosis, myocardial infarction, and aortic aneurysm [51].

In the setting of ischemic HF, NGAL serum levels are correlated with the functional status of the patient evaluated by the NYHA class [52] and can predict mortality independently of renal disease [53]. NGAL levels were shown to be significantly associated with coronary artery disease [54] and a larger amount of interstitial fibrosis after myocardial infarction [55]. Also, it has been shown that NGAL acts as a growth factor by promoting the proliferation of cardiac fibroblasts [56], promoted by aldosterone, leading to collagen I deposition and fibrosis [55].

Furthermore, high levels of NGAL are reported to be associated with frailty and cognitive impairment, independently of creatinine clearance, albuminuria, or other CKD risk factors [57].

**Figure 2:**
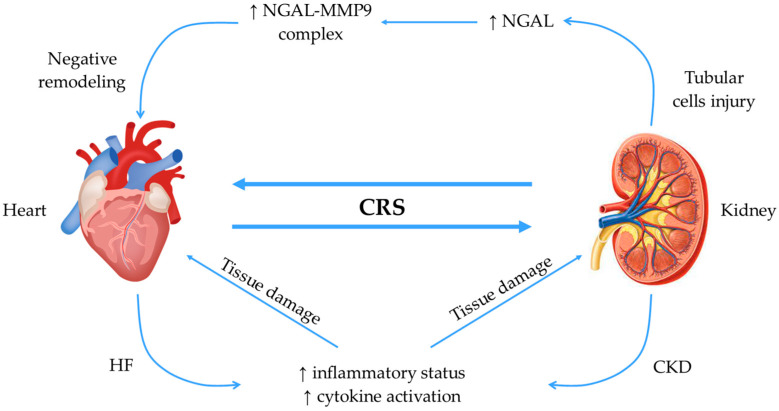
Mechanisms of action of NGAL. In CRS, there is a complex cross-talk between the heart and the kidney, and failure of each organ leads to an inflammatory status and cytokine activation. When a tubular injury occurs, secretion of NGAL increases, and it binds MMP9 to form a complex. This results in negative remodeling of the myocardium, which further amplifies the vicious circle. CKD, chronic kidney disease; CRS, cardio-renal syndrome; HF, heart failure; NGAL, neutrophil gelatinase-associated lipocalin; MMP9, matrix metalloproteinase 9.

The mechanisms of action of NGAL in the interaction between the heart and the kidney are summarized in Figure 2.

### 2.5. Relaxin

Relaxin is a peptide hormone that was initially known for its role during pregnancy, as it is involved in vasodilation, increased cardiac output, and remodeling of the ECM in the vagina, uterus, and pubic ligament [58]. It presents structural similarities with insulin and insulin growth factors, but despite these similarities, the families of relaxin and insulin are maintained separated because they detain major differences in receptors, signal pathways, and biological effects [59]. Relaxin is mainly produced in the corpus luteum in pregnant and non-pregnant women and achieves its highest plasma levels during pregnancy. In males, relaxin was found to be produced by the prostate with release in the seminal fluid. Human atria were also found to secrete relaxin [60].

Later, it was discovered that relaxin is responsible for other non-reproductive processes. Relaxin-null mice developed spontaneous fibrosis in multiple organs like skin, lung, kidney, and heart, and similar effects were observed in mice lacking the Relaxin Family Peptide Receptor 1 (RXFP-1) [58]. Relaxin effectively reduces renal fibrosis, with recent research indicating that relaxin suppresses TGF-β activity in human and rat kidney myofibroblast through its receptor RXFP1, activating a pathway dependent on neuronal NOS-NO-cGMP and extracellular signal-regulated kinase phosphorylation (pERK). This inhibition curtails TGF-β induces abnormal matrix/collagen production and myofibroblast differentiation while enhancing matrix metalloproteinases that degrade collagen. Moreover, relaxin diminishes Wnt/b-catenin signaling, thereby reducing epithelial-mesenchymal transition and cell cycle arrest in tubular epithelial cells, mitigating renal fibrosis. In addition, relaxin is capable of promoting ECM degradation by stimulating the MMP concomitant with the inhibition of their tissue inhibitors [61,62,63,64].

In heart disease, Pintalhao et al. found that in the setting of acute HF, relaxin serum levels are correlated with echocardiographic parameters of right heart overload, such as higher systolic pulmonary artery pressure, right ventricle dilation and dysfunction, right atrium dilation and reduced diameter variability with inspiration of the inferior vena cava. The authors found no differences between genders and no correlation between relaxin levels and age or renal impairment [65].

Relaxin also suppresses cardiac arrhythmias by various mechanisms. Henry et al. used aged rats known for their high prevalence of atrial fibrillation to study the effects of relaxin on this arrhythmia. On the one hand, the authors documented histologic evidence of reduction of atrial myocyte hypertrophy and reversal of atrial fibrosis. This can be explained at a molecular level by the inhibition of pro-fibrotic factors such as TGF-β1, collagen I and III, MMP, and reduced phosphorylation of connexin 43. On the other hand, a higher current density of voltage-gated sodium channels INa was observed [66]. Based on these data, we can conclude that relaxin possesses the ability to induce the remodeling of the myocardium both morphologically and electrically.

These findings underscore relaxin’s potential as a therapeutic intervention for renal fibrosis by targeting multiple pathways involved in fibrotic progression, which will be described in Section 3.2.

### 2.6. Collagen Biomarkers

Collagen is involved in cellular turnover, leading to tissue remodeling and fibrosis formation. Collagen types 1 and 3 are the predominant subtypes of collagen in the human body, and three biomarkers derived from their metabolism are shown to have potential value for the non-invasive assessment of organ fibrosis—Procollagen type I carboxy-terminal propeptide (PICP), carboxyterminal telopeptide of collagen type I (CITP), and Procollagen type III N-terminal peptide (P3NP) [67].

Initial data regarding collagen biomarkers in cardio-renal diseases refer to the role of PICP (involved in collagen type 1 formation) as an indicator of an active pro-fibrotic state. This biomarker can be used both for the diagnosis and prognosis of heart failure [68]. In patients with chronic kidney disease, PICP was associated with left ventricular filling pressures and predicted cardiovascular death and all-cause mortality [69]. A recent study from our group proved that in patients with advanced chronic kidney disease, not yet on dialysis, PICP is associated with the value of global longitudinal strain, suggesting subclinical fibrosis [70].

Robust data supports using CITP (an indicator of collagen type 1 degradation) as a biomarker, mostly in heart failure cohorts, for the diagnosis and prognosis of this disease [71,72]. Additionally, this marker seems to predict the risk of recurrence after atrial fibrillation ablation and the risk of defibrillator shocks in patients with an implanted cardiac defibrillator [73].

P3NP (an indicator of collagen type 3 synthesis) can help with the diagnosis of heart failure, atrial fibrillation, and hypertension as a prognostic marker in post-myocardial infarction and heart failure patients [72,74]. Data regarding its use in CKD are scarce, with studies suggesting a potential role for the urinary P3NP [75]. A study from our group proved that PICP and P3NP are good predictors of all-cause mortality in advanced chronic kidney disease patients not yet on dialysis [76].

Endotrophin, also known as the collagen VI alpha-3 chain, is another collagen-derived biomarker that has garnered attention for its role in cardio-renal fibrosis. Endotrophin is generated by proteolytic cleavage of collagen VI and plays a critical role in fibrosis by promoting fibroblast activation, inducing inflammation, and stimulating endothelial mesenchymal transition. Moreover, endotrophin interacts with the TGF-β signaling pathway, a key regulator of fibrosis. TGF-β induces the expression of endotrophin, while endotrophin, in turn, can potentiate TGF-β signaling, creating a positive feedback loop that amplifies fibrotic responses in the heart and kidneys [77,78].

Targeting collagen pathways and endotrophin, with their downstream pathways, may represent a promising therapeutic approach for managing fibrotic diseases of the heart and kidneys. However, further research is needed to elucidate the precise mechanisms of collagen-mediated fibrosis and to develop effective therapeutic interventions.

### 2.7. Other Mechanisms and Pathways

#### 2.7.1. Redox Signaling and Oxidative Stress

Redox signaling refers to the intricate interplay between reactive oxygen species (ROS) and antioxidant defense mechanisms, which impacts disease progression and pathogenesis.

In CKD and HF, an imbalance between ROS production and antioxidant capacity leads to oxidative stress, exacerbating renal damage. ROS, including superoxide anion, hydrogen peroxide, and hydroxyl radicals, serve as signaling molecules, regulating various cellular processes such as inflammation, apoptosis, and fibrosis [79].

Excessive ROS production arises from multiple sources, including mitochondrial dysfunction (a major contributor, as impaired electron transport chain activity leads to increased ROS production), inflammation (by activating NADPH oxidases), and RAAS activation (as described above). Oxidative stress damages cellular components, including DNA, proteins, and lipids. ROS can oxidize DNA bases, leading to mutations and genomic instability. Protein oxidation can impair enzyme function and disrupt cellular signaling pathways. Lipid peroxidation, initiated by a ROS attack on membrane lipids, compromises membrane integrity and alters cellular function [79,80].

Redox-sensitive signaling pathways are dysregulated in CKD and HF, contributing to inflammation, apoptosis, and fibrosis. Nuclear factor-κB (NF-κB) is a key transcription factor involved in inflammatory responses and is activated via the ROS, promoting the expression of pro-inflammatory cytokines and adhesion molecules. Mitogen-activated protein kinases (MAPKs), including extracellular signal-regulated kinase (ERK), c-Jun N-terminal kinase (JNK), and p38 MAPK, are activated via the ROS and regulate cell proliferation, apoptosis, and inflammation [80]. Conversely, endogenous antioxidant defense mechanisms counteract ROS-induced damage to maintain redox homeostasis. These include enzymes such as superoxide dismutase (SOD), catalase, glutathione peroxidase, and non-enzymatic antioxidants like glutathione and vitamins C and E [80].

Modulating redox-sensitive signaling pathways may offer therapeutic potential in CKD and HF by attenuating inflammation and fibrosis.

#### 2.7.2. Inflammasomes

Inflammasomes, critical innate immune system components, have emerged as significant contributors to cardio-renal fibrosis. They are activated by oxidative stress, tissue injury, and inflammation, promoting pro-inflammatory responses by facilitating the maturation and secretion of IL-1β and IL-18. Inflammasome activation involves the assembly of NOD-like receptor (NLR) family proteins, such as NLRP3, with the adaptor protein ASC (apoptosis-associated speck-like protein containing a CARD) and pro-caspase-1. This assembly leads to the activation of caspase-1, which subsequently cleaves pro-IL-1β and pro-IL-18 into their active forms, IL-1β and IL-18. Both IL-1β and IL-18 are potent pro-inflammatory cytokines capable of promoting fibrosis directly. They stimulate fibroblast activation and proliferation and induce the synthesis of ECM proteins, such as collagen, fibronectin, and elastin. Additionally, IL-1β and IL-18 can recruit inflammatory cells to the site of injury, further exacerbating the fibrotic response [81,82].

Inflammasome activation intersects with several other signaling pathways implicated in fibrosis. For example, the NLRP3 inflammasome can interact with the TGF-β signaling pathway, a master regulator of fibrosis. Activation of NLRP3 inflammasome can potentiate TGF-β-induced fibrotic responses, while TGF-β itself can induce NLRP3 expression, creating a positive feedback loop that amplifies fibrosis. Emerging evidence suggests that inflammasome activation may also influence epigenetic mechanisms involved in the regulation of fibrosis-related gene expression. For instance, inflammasome activation can modulate histone modifications and DNA methylation patterns, altering the accessibility of chromatin and influencing the transcriptional activity of fibrosis-associated genes [83,84].

These mechanisms have been proven in cardiac fibroblasts, endothelial cells, and renal tubular cells, pointing toward an important role of the inflammasome in cardio-renal fibrosis [82,84]. Overall, inflammasomes play a central role in orchestrating the inflammatory and fibrotic responses observed in cardio-renal diseases. Understanding the intricate molecular mechanisms underlying inflammasome activation and its downstream effects on fibrosis is essential for the development of targeted therapies aimed at mitigating fibrotic progression in these conditions.

## 3. Emerging Therapeutic Targets

### 3.1. RAAS Inhibitors—Future Perspectives

**Figure 3:**
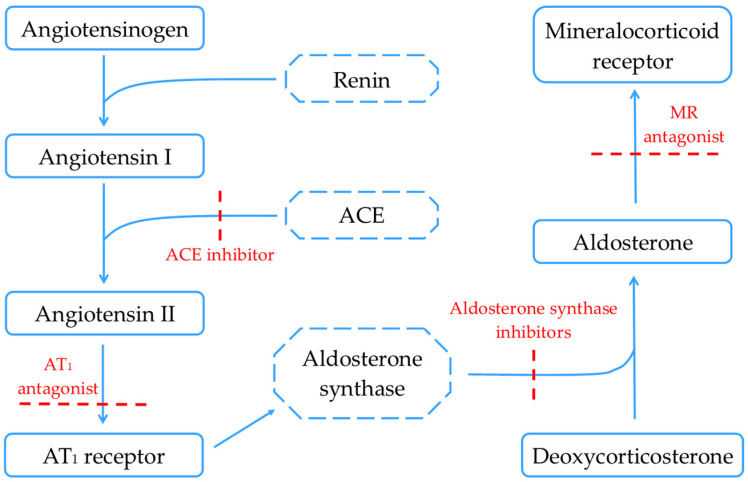
Pathways and inhibiting mechanisms of the renin–angiotensin–aldosterone system. ACE, angiotensin-converting enzyme; AT_1_, Angiotensin II receptor type 1; MR, mineralocorticoid receptor.

Various methods for inhibiting the RAAS in order to limit fibrosis have been proposed or are currently under evaluation to minimize the adverse effects and increase efficacy. These inhibiting mechanisms, which constitute targets for developing new therapies among the classic inhibitors (ACE inhibitors, AT1 antagonists, and MR antagonists), are presented in Figure 3.

Aldosterone synthesis inhibitors (e.g., Baxdrostat) are a new class of pharmaceutic agents with remarkable results in preclinical studies regarding their anti-remodeling properties. However, the low selectivity for the aldosterone synthase enzyme, epigenetic regulation, and alteration to the MR chaperone state currently limits them. Developing mechanisms to deliver the pharmacologic agent to selected targets (liposomes and antibodies) would help limit the risk of adverse effects [85]. Liu et al. discovered a range of isoindoline-1-one derivatives characterized by their potent and selective inhibition of aldosterone synthase, offering a promising foundation for the development of a new generation of highly specific aldosterone synthase inhibitors which would mark a critical advancement in the field [86].

Finerenone, an innovative selective non-steroidal MR antagonist, showed remarkable safety and efficacy in the FIDELIO-DKD and FIGARO-DKD trials in reducing both renal and cardiovascular complications in patients with type 2 diabetes and CKD [87,88]. Furthermore, a CONFIDENCE trial has recently been announced and aims to study finerenone associated with empagliflozin compared with each therapeutic agent alone in the setting of CKD and diabetes [89]. The results of the MIRACLE study are awaited regarding the efficacy and safety of the non-steroidal MR antagonist Balcinrenone in combination with dapagliflozin in patients with HF and CKD, compared with dapagliflozin alone [90]. Moreover, another recently developed molecule of the non-steroidal MR antagonist class is KBP-5074 [91].

For the time being, steroidal MR antagonists, such as spironolactone and eplerenone, will probably maintain their important role in treating cardiorenal diseases and limiting fibrosis and remodeling. As evidence continues to emerge, the new emerging classes of therapeutic agents could become important players in the field of cardiorenal diseases.

### 3.2. Serelaxin

Taking into account the effects and the anti-fibrotic properties of relaxin previously described, there has been developed the recombinant human relaxin hormone, also known as serelaxin [92], which allowed the conduction of various preclinical and clinical studies.

In mice, serelaxin showed potential nephroprotective effects by decreasing tumoral necrosis factor alpha (TNF-α) and IL-1α levels and suggesting an anti-inflammatory effect [93]. This effect is in part attributed to the inhibition of EMT via Notch1 signaling with the mediation of RXFP-1 [94].

In humans, the RELAX-HF trial was conducted on patients with acute heart failure and showed beneficial effects on dyspnea and all-cause and cardiovascular mortality at 180 days follow-up [95]. Another recent phase III trial failed to prove these results, and infusion of serelaxin as compared to placebo did not improve the incidence of all-cause or cardiovascular mortality, rehospitalization for heart failure or renal failure, or the length of hospitalization [96].

There are several reasons which may explain the contrasting results of these trials. Fibrosis is a chronic disease, by definition, and it might require long-term administration of serelaxin. However, serelaxin has a short half-life, around 2 h, with rapid loss of its in vivo effects, and this suggests the need for repeated daily doses [92]. Furthermore, concomitant medication such as angiotensin receptor-1 antagonist can limit the response of myofibroblasts to serelaxin and reduce its anti-fibrotic effect [97]. Lastly, it was shown that in patients with osteosarcoma, serelaxin increases the risk of metastasis; thus, long-term treatment should be avoided to prevent this risk from unknown malignancies [92].

### 3.3. Neutralizing Interleukin-11 (IL-11) Antibodies

IL-11 was recently discovered to be an important element in cardiac and renal fibroblast activation, leading to organ fibrosis. In mice, the overexpression of IL-11 led to heart and kidney fibrosis, while the genetic deletion of the IL-11 receptor (IL-11RA) protected against it [98]. As IL-11 is a downstream regulator of TGF-β in the pathway that culminates with tissue fibrosis, the idea that neutralizing antibodies anti-IL-11 or anti-IL-11RA appeared, and it returned positive results in preclinical studies by preventing or even reversing fibrosis in lung [99] and liver [100]. Schafer et al. proved that neutralizing IL-11 antibodies lowered the cardiac and renal fibrotic effect of TGF-β in murine models [101]. Recently, two studies have shown that IL11-induced mesenchymal transition of tubular cells represents a major step in renal pathology and that neutralization of IL11 improves tubular function by preventing this process [102,103].

Considering these results, the neutralizing IL-11 antibodies represent a potential therapeutic target for cardiorenal fibrosis, and further clinical studies are needed to establish its safety and efficacy.

### 3.4. Individualized Therapeutic Approach

Considering the various mechanisms contributing to fibrosis, the concept has emerged that distinct patient profiles could derive significant benefit from precisely tailored anti-fibrotic treatments, emphasizing the importance of personalized therapeutic approaches in clinical practice.

In this respect, the FIBROTARGETS program is a multinational academic and industrial consortium that seeks to identify new fibrotic targets and mechanisms for the development of specific therapies for heart failure through the investigation of circulating markers and the stratification of patients into distinct “fibrotic” bioprofiles. It aims to characterize key pathways in fibrillary collagen metabolism, evaluate potential anti-fibrotic molecules, and develop individualized diagnostic and therapeutic options by examining myocardial fibrosis mechanisms [104].

## 4. Conclusions

In conclusion, fibrosis plays a central role in the complex relationship between CVD and CKD as a cornerstone of the intricated molecular pathways, ultimately leading to organ dysfunction. The high burden of morbidity and mortality in this setting justifies the interest in a deeper understanding of the physiopathology of these conditions. Advanced comprehension of the involved mechanisms allows the development of novel therapeutic strategies, which are awaited to attest to their safety and efficacy.

## Data Availability

Not applicable.
